# Determining epigenetic memory in kidney proximal tubule cell derived induced pluripotent stem cells using a quadruple transgenic reprogrammable mouse

**DOI:** 10.1038/s41598-022-24581-z

**Published:** 2022-11-25

**Authors:** Gabriel Khelifi, Theresa Chow, Jennifer Whiteley, Victoire Fort, Benjamin D. Humphreys, Samer M.I. Hussein, Ian M. Rogers

**Affiliations:** 1 Cancer Research Center, Université Laval, Quebec City, QC Canada; 2 Oncology Division, CHU of Québec-Université Laval Research Center, Quebec City, QC Canada; 3 Lunenfeld Tanenbaum Research Institute, Mount Sinai Hospital, Toronto, ON Canada; 4 Department of Physiology, University of Toronto, Toronto, ON Canada; 5 Division of Nephrology, Department of Medicine, Department of Developmental Biology, Washington University in St. Louis School of Medicine, St. Louis, MO USA; 6 Department of Obstetrics and Gynaecology, University of Toronto, Toronto, ON Canada; 7 Ajmera Transplant Center, UHN, Toronto, Canada

**Keywords:** Cell biology, Computational biology and bioinformatics, Developmental biology, Stem cells

## Abstract

The majority of nucleated somatic cells can be reprogrammed to induced pluripotent stem cells (iPSCs). The process of reprogramming involves epigenetic remodelling to turn on pluripotency-associated genes and turn off lineage-specific genes. Some evidence shows that iPSCs retain epigenetic marks of their cell of origin and this “epigenetic memory” influences their differentiation potential, with a preference towards their cell of origin. Here, we reprogrammed proximal tubule cells (PTC) and tail tip fibroblasts (TTF), from a reprogrammable mouse to iPSCs and differentiated the iPSCs to renal progenitors to understand if epigenetic memory plays a role in renal differentiation. This model allowed us to eliminate experimental variability due to donor genetic differences and transfection of the reprogramming factors such as copy number and integration site. In this study we demonstrated that early passage PTC iPSCs and TTF iPSCs expressed low levels of renal progenitor genes and high levels of pluripotency-associated genes, and the transcriptional levels of these genes were not significantly different between PTC iPSCs and TTF iPSCs. We used ChIP-seq of H3K4me3, H3K27me3, H3K36me3 and global DNA methylation profiles of PTC iPSCs and TTF iPSCs to demonstrate that global epigenetic marks were not different between the cells from the two different sets of tissue samples. There were also no epigenetic differences observed when kidney developmental genes and pluripotency-associated genes were closely examined. We did observe that during differentiation to renal progenitor cells the PTC iPSC-derived renal cells expressed higher levels of three renal progenitor genes compared to progenitors derived from TTF iPSCs but the underlying DNA methylation and histone methylation patterns did not suggest an epigenetic memory basis for this.

## Introduction

Somatic cell reprogramming as described by Takahashi and Yamanaka in 2006 has opened up whole new areas of research into the genetic and epigenetic regulation of embryo development, stem cell differentiation, direct reprogramming, transdifferentiation and regenerative medicine^1^. Despite the many publications on reprogramming we still do not have a complete understanding of this process and still lack sufficient control over the finer details of reprogramming^2–4^. Experimental evidence indicates that cell of origin and donor differences could impact the differentiation capabilities of the final induced pluripotent stem cell (iPSC) line.

Intensive epigenetic remodelling occurs during cellular reprogramming of somatic cells to iPSCs to allow endogenous pluripotency-associated genes to turn on and mature genes to turn off. Studies have demonstrated that cell of origin epigenetic signatures can persist and influence the differentiation propensity of the iPSCs.

The first studies demonstrating epigenetic memory were published in 2010. Since then multiple labs have demonstrated that early passage iPSCs (p < 20) have been shown to have an epigenetic signature that resembles their cell of origin^5–8^. For instance, Kim and colleagues demonstrated that blood derived iPSCs have a DNA methylation pattern more similar to blood cells and a higher propensity to differentiate and form blood colonies than fibroblast derived iPSCs^6^. Bar-Nur and colleagues showed that iPSCs derived from pancreatic beta cells could differentiate into insulin-secreting cells more easily than embryonic stem cells (ESCs) or iPSCs derived from non-beta pancreatic cells^5^.

Kidney disease affects millions of people worldwide and is a good candidate for iPSC-based therapies, as dialysis provides time to develop a personalized cell therapy strategy. Renal cells can easily be collected from the urine and reprogrammed into iPSCs using clinically relevant reprogramming vectors^9^. Despite the affinity of renal disease to be treated with iPSC-based therapies there are no studies investigating epigenetic memory of renal cells.

Epigenetic changes caused by chemical modifications to the chromatin, such as methylation and acetylation, as opposed to changes to the genomic sequence are considered the basis for a greater potential of an iPSC line to differentiate back to its cell of origin. DNA methylation status affects chromatin condensation, with highly methylated DNA near the transcription start site (TSS) being indicative of inactive genes^10^. On the other hand, the effect of histone modifications on gene expression is combinatorial and site specific. Histone H3 lysine 4 trimethylation (H3K4me3) marks transcriptionally active genes while Histone H3 lysine 27 trimethylation (H3K27me3) and Histone H3 lysine 9 dimethylation (H3K9me2) mark transcriptionally repressed genes^11,12^. Bivalent chromatin poised for activation is marked with both H3K4me3 and H3K27me3^13,14^. Histone H3 lysine 36 trimethylation (H3K36me3) coincides with H3K4me3 and marks transcriptionally active genomic regions with a role in transcription extension^15,16^.

Given the importance of epigenetic memory on differentiation capacity, we sought to investigate the possible influence of epigenetic memory on the differentiation of renal cell derived iPSCs to renal progenitors. In this report we focused on the impact cell of origin epigenetics has on the reprogramming and differentiation capacity of iPSC lines derived from renal proximal tubule cells. We hypothesized that proximal tubule cell iPSCs (PTC iPSCs) would retain epigenetic and transcriptional memory of their cell of origin and this memory would allow them to differentiate to renal progenitors more readily than tail tip fibroblast iPSCs (TTF iPSC). To test this hypothesis, we made PTC iPSCs and TTF iPSCs from a novel reprogrammable mouse.

To eliminate confounding factors attributed to the donor genome and reprogramming procedure, we produced a quadruple transgenic mouse to unequivocally test the effect of epigenetic memory.

The reprogrammable mouse cells all possess the same copy of the reprogramming transgenes, integrated in the same genomic location. The quadruple transgenic mouse also contains a kidney specific Cre-reporter that allowed us to isolate kidney proximal tubule cells with certainty due to a GFP/tdTomato reporter system under the control of the PTC specific gene SCL34a1 promoter. It is important to pre-mark proximal tubule cells before isolation and in vitro culture because the PTC will lose surface proteins during culture making them more difficult to identify. This strategy also eliminates the chances of reprogramming a mesangial cell or endothelial cell. Comparatively, tail tip fibroblasts can be cultured in vitro in appropriate media for up to 10 passages without change to their identity, thus pre-isolation tagging is not required. Overall, this quadruple transgenic mouse model with a ‘controlled reprogrammable system’ allowed us to determine if renal cell-specific epigenetic marks were retained during reprogramming and influenced the efficacy of differentiation in a cell of origin-specific manner.

We demonstrated, using reverse-transcription quantitative polymerase chain reaction (RT-qPCR) that the expression of renal progenitor genes was low and not significantly different between undifferentiated PTC iPSCs and TTF iPSCs indicating complete reprogramming and no residual cell of origin expression. ChIP-seq and long read DNA-methylation sequencing demonstrated that early passage (p < 10) PTC iPSC and TTF iPSCs had similar global DNA methylation and histone methylation patterns indicating an equivalence in reprogramming. Extensive examination of the global epigenetic status of sets of genes linked to differentiation to specific organs and tissue including kidney revealed no differences between PTC-iPSCs and TTF-iPSCs, Despite the lack of epigenetic memory we did observe higher gene expression for three renal genes during differentiation of the PTC iPSCs compared to the TTF iPSCs. However, we could not link this finding to any epigenetic memory retained from the cell of origin. Our results suggest that early passage PTC iPSCs do not exhibit epigenetic memory as defined by unique histone methylation or DNA methylation at kidney-associated genes.

## Materials and methods

### Ethics statement

Experiments, using only mouse cells, were carried out at the Lunenfeld Tanenbaum Research Institute at Mount Sinai Hospital in Toronto, Canada. Animal use protocol was approved by Lunenfeld Tanenbaum Research Institute at Mount Sinai Hospital in Toronto, Canada.

No other animals or human subjects/tissues were used.

### Breeding and genotyping mice

SLC34a1-GFPCreERt2 (SLC34a1-GCE) mice (courtesy of Dr. Benjamin Humphreys) were re-derived at the Toronto Centre for Phenogenomics (Toronto, ON) and bred with tdTomato mice (B6;129S6-Gt(ROSA)26Sor^tm14(CAG-tdTomato)Hze^/J) to produce SLC34a1-GCE; tdTomato double transgenic mice^17^. SLC34a1-GCE; tdTomato were then bred with rtTAROSA (neo-in); OKMS-250 mice (courtesy of Dr. Andras Nagy) to generate the SLC34a1-GCE; tdTomato; rtTAROSA (neo-in); OKMS-250 mouse line. In these mice, every cell has doxycycline (DOX)-inducible reprogramming factors (Oct4, Klf4, c-Myc and Sox2) and mature proximal tubule cells (PTC) expressed SLC34a1 and Cre recombinase. As illustrated in Supplementary Fig. 1a, upon tamoxifen treatment the Cre recombinase excises the loxP-flanked neo cassettes on the tdTomato and rtTA-ROSA transgenes, which enables PTC to fluoresce red. GFP from the SLC34a1-GCE construct helps to confirm the cells contain the SLC34a1-GCE construct and the presence of tdTomato after tamoxifen induction confirms the CRE was activated due to the SCL4a1 promoter thus labeling the PTC red (and green).

The SLC34a1-CRE also activates the rtTA in the ROSA locus allowing the activation of the reprogramming factors upon the addition of Doxycycline. The addition of DOX allows PTC to be reprogrammed to induced pluripotent stem cells (iPSCs). For cells that do not express SLC34a1, there is no CRE and the cells cannot be reprogrammed ensuring that only PTC are reprogrammed (Supplementary Fig. 1b,c).

We tested the rtTAROSA (neo-in); OKMS-250 mouse cells prior to breeding with the SLC34a1-GCE; tdTomato mice to determine if mCherry expression was leaky and could interfere with isolating tdTomato red cells later on. We were able to demonstrate that without the addition of DOX the mCherry remains off. rtTAROSA (neo-in); OKMS-250 mouse cells were treated with 1.5 µg/ml Dox and mCherry is visible by fluorescent microscopy. Cells were also tested by flow cytometry. Dox+ cells, Dox− cells and wild type cells were analyzed (Supplementary Fig. 1d–g).

In order to induce reprogramming in the tail tip fibroblasts from the quadruple transgenic mice, exogenous Cre recombinase was introduced to remove the loxP-flanked neo cassettes to facilitate iPSC reprogramming. Pups were weaned and genotyped at 3 weeks old. Ear clips were digested in 100 µl of 0.05 M NaOH for 10 min at 98 °C, and the solution was neutralized with 10 µl of 1 M TRIS, pH 8.0. Genomic DNA was then diluted with 100 µl of molecular grade sterile water. One microliter of DNA sample was used in PCR. PCR conditions for ROSA rtTA primers: 94 °C for 3 min followed by 29 cycles of 94 °C for 30 s, 56 °C for 30 s, 72 °C for 30 s, and a final extension at 72 °C for 5 min. PCR conditions for SLC34a1, tdTomato, and OKMS-250 primers: 94 °C for 3 min followed by 30 s cycles of 94 °C for 30 s, 60 °C for 30 s, 72 °C for 30 s, and a final extension at 72 °C for 5 min. Only heterozygous mice positive for all four transgenes were used for experiments. Genotyping primers: SLC34a1 Cre (F: TTGCCTGCATTACCGGTCGATGCAACGAGT, R: CCTGGTCGAAATCAGTGCGTTCGAACGCTA), SLC34a1 WT (F: ACAAAACCCTACTGGGTGGA, R: CTCGCTGTAGGACATCAT), tdTomato mutant (F: CTGTTCCTGTACGGCATGG, R: GGCATTAAAGCAGCGTATCC), tdTomato WT (F: AAGGGAGCTGCAGTGGAGTA, R: CCGAAAATCTGTGGGAAGTC), ROSA rtTA mutant (F: AAAGTCGCTCTGAGTTGTTAT, R: GCGAAGAGTTTGTCCTCAACC), ROSA rtTA WT (F: AAAGTCGCTCTGAGTTGTTAT, R: GGAGCGGGAGAAATGGATATG), OKMS-250 mutant (F: GTGCCAAAGTTGTTCTGACTG, R: AGGGGGCAATCCATTTTCTTT), OKMS-250 WT (F: TCGTACCCTCATTCCCTCTG, R: AGGGGGCAATCCATTTTCTTT).

### Tamoxifen treatment

To induce Cre-mediated recombination in proximal tubule cells, 6-week-old SLC34a1-GCE; tdTomato; rtTAROSA (neo-in); OKMS-250 mice were fed tamoxifen food pellets (Envigo, TD.130858) for 2 weeks. Mice were euthanized at 8–10 weeks using CO_2_, and kidneys and tail tip were collected from the mice.

### Isolating and reprogramming proximal tubule cells (PTC) to induced pluripotent stem cells

Kidney proximal tubules were isolated and cultured as previously described^18^. Briefly, the kidney cortex was excised and minced into 1–3 mm^3^ pieces. The cortex pieces were then digested in 0.5 mg/ml collagenase (Sigma, P9891) for 30 min at 37 °C. During digestion, the cortex pieces were gently agitated every 10 min by pipetting up and down. After 30 min, fetal bovine serum (FBS) was added to stop the digestion. The digested tissue was then passed through a 70 µm cell strainer to filter out the glomeruli, and centrifuged at 400*g*, 10 °C for 5 min to pellet the tubules. Finally, tubules were cultured in DMEM/F12 supplemented with 10 µg/ml insulin, 5.5 µg/ml transferrin, and 5 µg/l selenium (ITS; Sigma, I-3146), 50 nM hydrocortisone (Sigma, H6909-10ML), and 50 units/ml penicillin and 50 µg/ml streptomycin^18^. Tubule cell outgrowth was observed 7–10 days post isolation.

Once cells grew to confluency, they were dissociated into single cells and tdTomato+/GFP+ cells were sorted for by fluorescence-activated cell sorting (FACS) (MoFlo Astrios EQ Cell Sorter, Beckman Coulter). Sorted tdTomato+/GFP+ cells were then cultured in 15% FBS Complete Media (DMEM containing 15% FBS, 50 units/ml penicillin, 50 µg/ml streptomycin, 2 mM GlutaMAX, 0.1 mM non-essential amino acids (NEAA), 1 mM sodium pyruvate, and 0.1 mM 2-mercaptoethanol) supplemented with 1000 units/ml leukemia inhibiting factor (LIF) and 1.5 µg/ml doxycycline (DOX) to initiate the reprogramming process. Induced pluripotent stem cell colonies appeared as early as 7 days post induction. Clonal lines were picked and expanded. DOX-independent clonal lines were established by gradually removing DOX from culture. DOX was kept on for 14 days then reduced to 1.0, 0.75 and 0.50 µm/ml every 2 days then to zero. Only DOX-independent lines were used for differentiation experiments.

### Isolating and reprogramming tail tip fibroblasts (TTF) to induced pluripotent stem cells

The tail tips of SLC34a1-GCE; tdTomato; rtTAROSA (neo-in); OKMS-250 mice were excised, cleaned with 70% ethanol, and cut into 1–3 mm^3^ pieces. The tail tip pieces were transferred onto plastic tissue culture plates and grown in 15% FBS Complete Media (DMEM containing 15% FBS, 50 units/ml penicillin, 50 µg/ml streptomycin, 2 mM GlutaMAX, 0.1 mM NEAA, 1 mM sodium pyruvate, and 0.1 mM 2-mercaptoethanol). Cell outgrowth appeared approximately 2–3 weeks after initial plating. Tail tip fibroblasts were grown to confluency and dissociated into single cells for electroporation. Non-integrating Cre episomal plasmids were introduced into TTF using the Neon electroporation system (Thermo Fisher) using the following parameters: 1200 mV, 2 ms, 2 pulses. Transfected cells were then cultured in 15% FBS Complete Media supplemented with 1000 units/ml LIF and 1.5 µg/ml doxycycline (DOX) to initiate the reprogramming process. DOX was kept on for 14 days then reduced to 1.0, 0.75 and 0.50 µm/ml every 2 days then to zero. Induced pluripotent stem cell colonies appeared approximately 14 days after transfection. Clonal lines were picked and expanded. Only DOX-independent lines were used for differentiation experiments.

### Immunocytochemistry

Immunocytochemistry was done as previously described^19^. The cell culture medium was removed and cells washed in phosphate buffered saline (PBS). 10% neutral buffered formalin (NBF) was used for 20 min at room temperature (RT) to fix the cells, followed by a second PBS wash containing 0.1% Triton X-100. Blocking of non-specific sites was accomplished by incubating with blocking buffer (PBS containing 0.1% Triton X-100 and 10% FBS) for 30 min at RT followed by the addition of primary antibodies (diluted 1:200) incubated overnight at 4 °C. Following this the cells were washed 4 × 10 min with PBS/0.1% Triton X-100 then the secondary antibodies (diluted 1:500) were added for 30 min in the dark and at RT. Cells were washed 5 × 10 min with PBS containing 0.1% Triton X-100, stained with DAPI and overlaid with PBS containing 50% glycerol, in the dark at 4 °C until imaging. Secondary antibody controls in which only the secondary antibodies were added were performed to confirm the specificity of the secondary antibody. Primary antibodies: Oct4 (Thermo Fisher, PA5-27438), Six2 (ProteinTech, 11562-1-AP), Wt1 (Abcam, AB89901), Pax2 (Biolegend, 901001). Secondary antibodies: anti-rabbit IgG Cy5 and anti-mouse IgG 488.

### Teratoma formation assay

Four PTC iPSC and four TTF iPSC lines were assessed for their ability to form teratoma. Induced pluripotent stem cells were grown under standard conditions until approximately 70–80% confluent. Cells were dissociated into single cells, and 4–8.5 million cells resuspended in Matrigel HC (Corning) were injected into 6–8 week old male NOD/SCID mice (Charles River). Teratomas were collected, fixed in 10% neutral buffered formalin (NBF), dehydrated and embedded in paraffin blocks. Teratoma analysis and interpretation were performed by a trained pathologist at The Centre for Phenogenomics (Toronto, ON).

### Hematoxylin and eosin staining

Paraffin-embedded tissues were sectioned, de-paraffinized, and rehydrated. Slides were stained with hematoxylin for 3 min followed by a 5 min wash with water. Next, slides were stained with eosin for 30 s followed by a series of washes: 95% ethanol for three washes (5 min each), 100% ethanol for three washes (5 min each), and xylene for three washes (15 min each). Slides were then mounted with DPX and cover-slipped.

### Reverse-transcription quantitative polymerase chain reaction (RT-qPCR)

RNA was isolated using the RNeasy Mini Kit (Qiagen), and complementary DNA (cDNA) was synthesized using Superscript II Reverse Transcriptase (Thermo Fisher) and amplified using the SensiFAST SYBR No ROX Kit (Bioline) under the following conditions: 95 °C for 15 s followed by 40 cycles of 95 °C for 5 s, and 60 °C for 30 s. Primers used: Pax2 (F: AAGCCCGGAGTGATTGGTG, R: CAGGCGAACATAGTCGGGTT), Six2 (F: CACCTCCACAAGAATGAAAGCG, R: CTCCGCCTCGATGTAGTGC), Cited1 (F: AACCTTGGAGTGAAGGATCGC, R: GTAGGAGAGCCTATTGGAGATGT), Sall1 (F: CTCAACATTTCCAATCCGACCC, R: GGCATCCTTGCTCTTAGTGGG), Wt1 (F: GAGAGCCAGCCTACCATCC, R: GGGTCCTCGTGTTTGAAGGAA), Hoxb7 (F: AAGTTCGGTTTTCGCTCCAGG, R: ACACCCCGGAGAGGTTCTG), Gata3 (F: CTCGGCCATTCGTACATGGAA, R: GGATACCTCTGCACCGTAGC), Foxd1 (F: CGCTAAGAATCCGCTGGTGAAG, R: GGATCTTGACGAAGCAGTCGTT), Pbx1 (F: CAGCGGGTTCTTCCAGTTCTT, R: CGAGTCCGTCACTGTATCCTC), CD34 (F: AAGGCTGGGTGAAGACCCTTA, R: TGAATGGCCGTTTCTGGAAGT), Jag1 (F: CCTCGGGTCAGTTTGAGCTG, R: CCTTGAGGCACACTTTGAAGTA), Cadherin 6 (F: CAGCCCTACCCAACTTTCTCA, R: GAACGGCTCAGCTCATTCC), Beta actin (F: GGCTGTATTCCCCTCCATCG, R: CCAGTTGGTAACAATGCCATGT), Dnmt3b (F: AGCGGGTATGAGGAGTGCAT, R: GGGAGCATCCTTCGTGTCTG), and Lin28a (F: GGCATCTGTAAGTGGTTCAACG, R: CCCTCCTTGAGGCTTCGGA).

Quantitative PCR data was acquired and analyzed using the Biorad CFX system. Expression data is displayed as the average of four cell lines, three replicates each and with standard deviation. Statistical significance between sample groups was calculated using *t* test and p values < 0.05 were considered significant.

### Differentiating mouse proximal tubule cell induced pluripotent stem cells and tail tip fibroblast induced pluripotent stem cells to renal progenitors

Four doxycycline-independent PTC iPSC lines and four doxycycline-independent TTF iPSC lines were differentiated to renal progenitor cells. Prior to differentiation, PTC iPSC and TTF iPSC were maintained on mitomycin C-treated mouse embryonic fibroblasts (MEF) and cultured in standard mouse embryonic stem cell media composed of DMEM supplemented with 15% FBS, 50 units/ml penicillin, 50 µg/ml streptomycin, 2 mM GlutaMAX, 0.1 mM non-essential amino acids (NEAA), 1 mM sodium pyruvate, 0.1 mM 2-mercaptoethanol and 100 units/ml leukemia inhibiting factor (LIF). For differentiation, PTC iPSC and TTF iPSC were dissociated into single cells using TrypLE (Invitrogen) and seeded on gelatin-coated tissue culture plates at a cell density of 25,000 cells/cm^2^. Cells were cultured in a series of induction media: Priming, Mesoderm, Intermediate Mesoderm, and Progenitor Induction media for two, two, two and 15 days, respectively^19^. Priming media: DMEM/F12 supplemented with 1 × N2 supplement, 1 × B27 supplement, 0.1 mM NEAA, 2 mM GlutaMAX, 50 units/ml penicillin, 50 µg/ml streptomycin, 12 ng/ml Fgf2, and 20 ng/ml Activin A. Mesoderm induction media: DMEM/F12 supplemented with 4% knockout serum replacement (KSR), 0.1 mM NEAA, 2 mM GlutaMAX, 50 units/ml penicillin, 50 µg/ml streptomycin, 1 mM sodium pyruvate, 0.015% w/v sodium bicarbonate, 0.1 mM 2-mercaptoethanol, 30 ng/ml Bmp4, 10 ng/ml Activin A, and 12 ng/ml Fgf2. Intermediate mesoderm induction was modified from Sambi and colleagues: DMEM/F12 supplemented with 4% KSR, 50 units/ml penicillin, 50 µg/ml streptomycin, 10 µM Y27632, 100 nM Retinoic acid, and 30 ng/ml Activin A^20^. Progenitor induction media was adapted from Kang and colleagues: RPMI-1640 supplemented with 1 × B27 supplement, 2 mM GlutaMAX, 50 units/ml penicillin, 50 µg/ml streptomycin, 150 ng/ml Bmp7, and 50 ng/ml Fgf2^21^. Only passage three to eight iPSCs were used for differentiation.

### Chromatin immunoprecipitation and sequencing (ChIP-seq)

Chromatin immunoprecipitation was performed on two PTC iPSC lines and two TTF iPSC lines (all in duplicates) following the protocol outlined in the SimpleChIP Enzymatic Chromatin IP Kit (Cell Signaling Technologies, 9003). Briefly, 30–60 million cells were fixed in 1% methanol-free formaldehyde (Thermo Fisher, 28906) at room temperature (RT) for 10 min. Glycine was added to the fixed cells at a final concentration of 0.125 M for 5 min at RT. Cells were centrifuged at 500*g*, 4 °C, 5 min, and washed twice with ice cold phosphate buffered saline (PBS). Chromatin was digested with micrococcal nuclease at a concentration of 0.1 µl/million cells for 30 min at 37 °C. Cells were then sonicated using the Sonics VibraCell (VCX130) with 3 mm probe at 40% amplitude and 5 sets of 20 s pulses. Approximately 10 µg of digested, cross-linked chromatin was incubated with 10 µg of anti-H3K4me3 (Abcam, AB8580, lot #GR3175719-3), anti-H3K27me3 (Millipore, 07-449, lot #3018864), or anti-H3K36me3 (Abcam, AB9050, lot #GR3210077-1) antibody overnight at 4 °C. ChIP-grade protein G magnetic beads were added to each immunoprecipitation reaction and incubated for 2 h at 4 °C. Magnetic beads were pelleted using a magnetic separation rack (Cell Signaling Technologies, 7017) and washed in a sequence of low salt and high salt washes. Finally, chromatin was eluted from the magnetic beads and DNA was reverse cross-linked from the histones. DNA was cleaned up using the Qiaquick PCR Purification Kit (Qiagen).

Enrichment was validated using quantitative PCR with Pax2 primers (Active Motif, #71020) and Actb2 primers (Active Motif, #71017). DNA was sequenced at The Centre for Applied Genomics (Toronto, ON) on the Illumina HiSeq 2500 and 50 million reads were acquired for each sample. The quality of the sequence data was assessed using FastQC (Version 0.11.5). Adaptors were trimmed using Trim Galore (Version 0.0.4).

### Processing and alignment of ChIP-seq data to identify H3K4me3, H3K27me3 and H3K36me3 enriched peaks

ChIP-seq sequencing data were processed using the Illumina analysis pipeline and FastQ format reads were aligned to the mm10 mouse reference using the Bowtie alignment algorithm^22^. Bowtie version 2.1.0 was used with the pre-set sensitive parameter to align ChIP sequencing reads (refer to ‘ChIP sequencing analysis pipeline’ below for more details).

#### Peak calling algorithm

The MACS version 2.0.10 (model based analysis of ChIPseq) peak finding algorithm was used to identify regions of ChIP-Seq enrichment over background^23^. Default parameters were used for H3K4me3, and broad peak parameters were used for H3K27me3 and H3K36me3 data (refer to ‘ChIP sequencing analysis pipeline’ below for details).

#### Peak annotation and processing

Multicov command from Bedtools v2.17.0 was used to obtain raw read counts within each histone mark peak identified by MACS and input reads within these peaks^24^. The number of reads per kilobase of peak per million reads (RPKM) was calculated for each peak and the corresponding input levels of that peak. The RPKM values for the histone mark peak were then subtracted by those of the input RPKM values to obtain a final and background-adjusted RPKM value, as modified from Hawkins et al*.*^25^. Peak calls with background-adjusted RPKM values less than or equal to 0 were excluded from further analysis. The background adjusted RPKM values were averaged across − 2 kb to + 3 kb of a gene transcription start site (TSS), as determined above, for downstream data analysis and visualization. Read densities and enrichment scores per locus, where defined, were normalized to the total number of million uniquely mapped reads producing values in units of reads per million mapped reads (RPM).

#### ChIP sequencing analysis pipeline

As described previously^3^, the sequences were trimmed to filter out the 3’ adaptor and remove last 2 bases and 3 extra bases if it matches with the adaptor sequence. Using Bowtie the sequences were mapped to the mouse genome (mm10) using the following sequence; (i) command: bowtie2 -p 8 –sensitive -x mm10/mm10 sequence.reads.fastq –S sample.sam (refer to Supplementary Table 1: 10.6084/m9.figshare.20368902). Peak calling algorithm MACS: (i) command for H3K4me3: macs2 callpeak -t chromatin.mark.file.bam -c input.sample.file.bam -f BAM -g mm -n [directory] –nomodel –shiftsize 73 –B; (ii) command for H3K27me3 and H3K36me3: macs2 callpeak –t chromatin.mark.file.bam –c input.sample.file.bam –broad –f BAM -g mm -n [directory] –nomodel –shiftsize 73 –B. Normalize unique mapped read values to library size. Annotate peaks to mouse genome (mm10).

### Long-read DNA sequencing and methylation analysis

#### gDNA library preparation

Extraction of genomic DNA from cells was done using the DNeasy Blood and Tissue kit from Qiagen according to the manufacture's instructions. Extracted gDNA was quantified using the Nanodrop reader and gel electrophoresis.

gDNA was fragmented by sonication with a Bioruptor sonicator for 30 cycles of 5 s ON–90 s OFF, using low intensity settings. gDNA was then purified with the Zymo DNA Clean & Concentrator™-5 columns (Zymo Research, D4013) and size selected with 0.4 × volume of KAPA HyperPure Beads (Roche, 08963835001) to exclude small DNA fragments. 1 μg of sheared gDNA was used for library preparation using Ligation Sequencing Kits (Oxford Nanopore Technologies, LSK109 and LSK110) according to the manufacturer’s specifications. Samples were sequenced on a MinION sequencer on R9 Flow Cells (Oxford Nanopore Technologies FLO-MIN106D), using the MinKNOW software v19. Flowcells were washed and reloaded with the Flow Cell Wash Kit (Oxford Nanopore Technologies EXP-WSH003) to increase sequencing depth obtained per flowcell. Average read N50 for sequencing runs was 3 kb.

#### Base calling and methylation analysis

Raw fast5 files were base called using the Guppy v.4.2.2 software, with the guppy_basecaller command and the dna_r9.4.1_450bps_modbases_dam-dcm-cpg_hac.cfg configuration file. GPU basecalling mode was employed with the following arguments: --num_callers 6 --gpu_runners_per_device 6 --chunks_per_runner 1000 --chunk_size 1000. Output fast5 files were generated with the --fast5_out argument. The resulting FASTQ reads were concatenated and then indexed with Nanopolish version 0.13.2^26^ using the nanopolish index command. Reads were aligned to the mm10 mouse reference with Minimap2 version 2.17-r941^27^. Using the following parameters: -a -x map-ont. BAM files were generated with SAMtools version 1.3.1^28^ and CpG methylation was called using the call-methylation function of Nanopolish. Methylation frequency for CpGs was analyzed using the calculate_methylation_frequency.py script from Nanopolish. The fraction of methylated CpGs at gene TSS was calculated with a sliding window approach of 300 bp, sliding every 30 bp. Analysis was confined to − 1 kb to + 1 kb region of TSS as we found this to be the region frequently spanning hypomethylation for key genes in pluripotent stem cells (refer to Supplementary Table 2: 10.6084/m9.figshare.368902). To correct for sample-to-sample differences, values for fraction methylated CpGs were first subtracted to distribution means in each separate samples, and then averaged for two independent animal replicates.

### Flow cytometry

Proximal tubule cells were isolated from rtTAROSA (neo-out); OKMS-250 mice and grown to confluency. Doxycycline (1.5 µg/ml) was added for 48 h prior to flow cytometry. On the day of flow cytometry analysis, cells were washed with Hanks’ Balanced Salt Solution (HBSS), dissociated into single cells using TrypLE Express (Invitrogen) and stained with DAPI at a concentration of 0.4 µg/ml. Cells were analyzed on the Gallios Flow Cytometer (Beckman Coulter Life Sciences) and with the Kaluza program.

### Statistical analyses

Two-tailed Student’s *t* test was used for comparisons of two samples together. Comparison of more than two samples was done with ANOVA followed by Tukey’s honest significant difference test. *P < 0.05, **P < 0.01, ***P < 0.001, ****P < 0.0001, *n.s.* not significant.

## Results

### Reprogramming proximal tubule cells (PTC) and tail tip fibroblasts (TTF) to induced pluripotent stem cells

**Figure 1:**
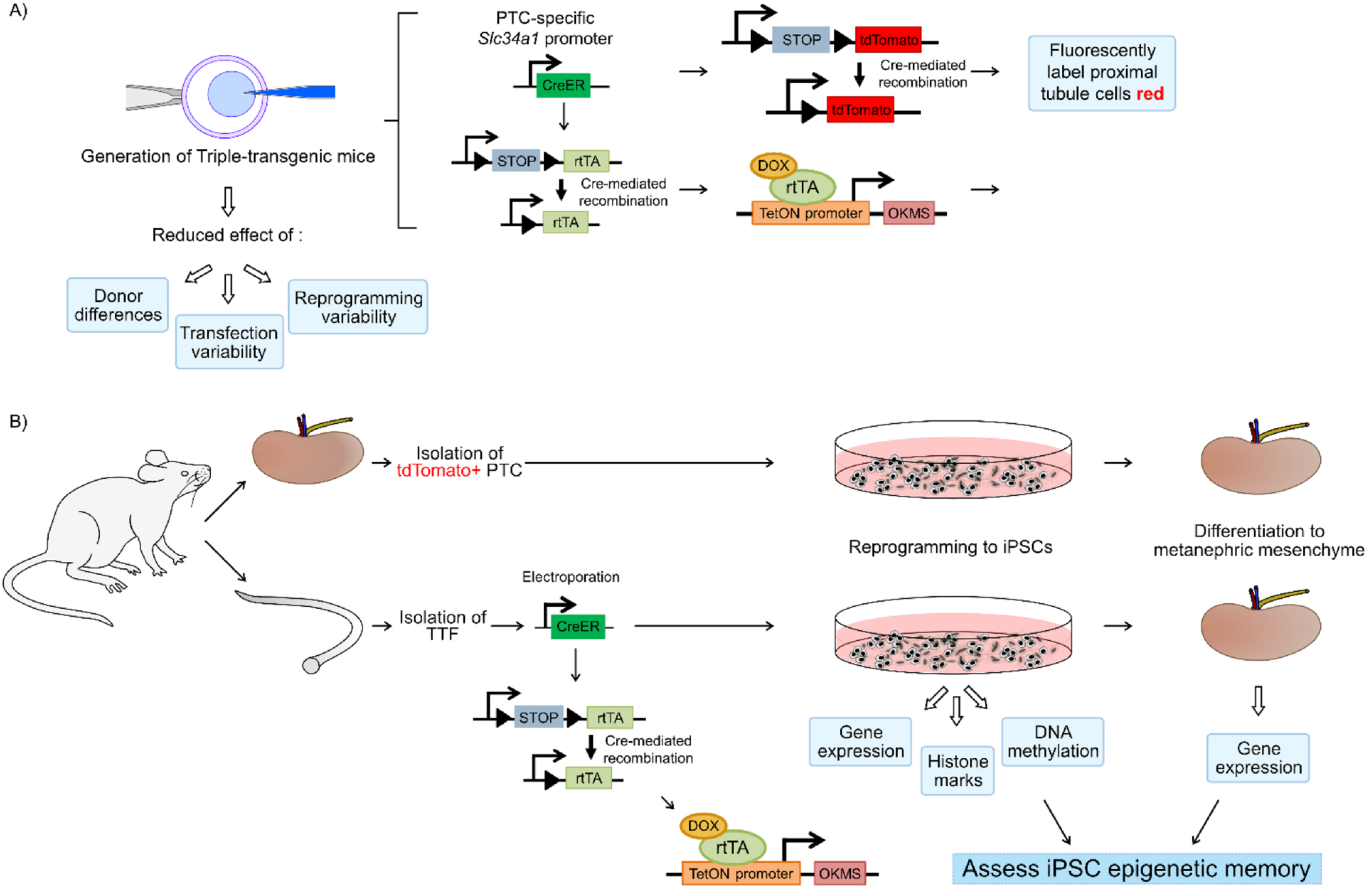
Research strategy overview. (**A**) SLC34a1-GFPCreERt2 (SLC34a1-GCE) mice were bred with tdTomato mice (B6;129S6-Gt(ROSA)26Sor^tm14(CAG-tdTomato)Hze^/J). These mice were bred to rtTAROSA (neo-in); OKMS-250 mice to generate the SLC34a1-GCE; tdTomato; rtTAROSA (neo-in); OKMS-250 mouse line. (**B**) Kidney proximal tubules and tail tip fibroblasts were isolated from the transgenic mice. Non-integrating Cre episomal plasmids were introduced into TTF to remove the loxP-flanked neo cassettes to facilitate iPSC reprogramming. To induce reprogramming the medium was supplemented with DOX. Gene expression, assessment of histone and DNA modification was done on the resultant iPSC lines. The iPSCs were also differentiated to renal progenitor and assessed.

To test the effect of epigenetic memory on iPSCs under well-controlled parameters where the same donor genome and reprogramming technique was used a quadruple transgenic mouse was produced. An overview of our research strategy is found in Fig. 1.

Proximal tubule cells (PTCs) and tail tip fibroblasts (TTF) were isolated from littermates. GFP+/Red fluorescent (td-Tomato)+ labelled PTCs were purified using fluorescence-activated cell sorting (FACS). An average of 29% tdTomato+/GFP+ PTC were isolated from kidneys (Supplementary Fig. 2).

**Figure 2:**
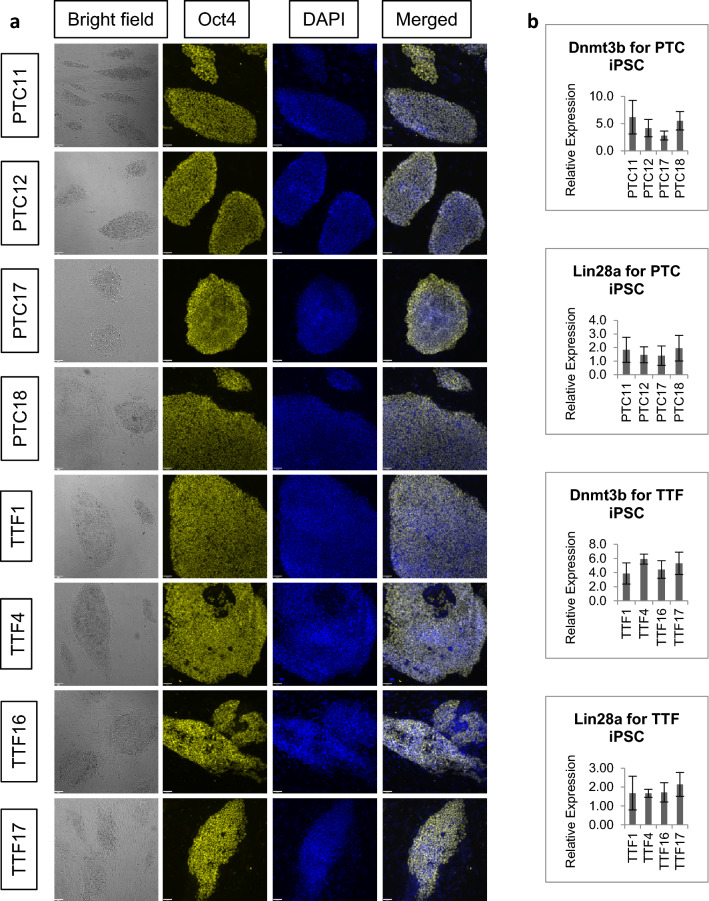
Expression of Oct4, Dnmt3b, and Lin28a in proximal tubule cell induced pluripotent stem cell (PTC iPSC) and tail tip fibroblast (TTF) iPSC. (**a**) Bright field microscopy shows that PTC iPSCs and TTF iPSCs display typical compact, dome-shaped iPSC colony morphology and immunocytochemistry shows that they express pluripotency-associated marker Oct4. Scale bar = 81 µm (bright field) and 40 µm (fluorescence). (**b**) RT-qPCR shows that PTC iPSC and TTF iPSC express pluripotency-associated markers Dnmt3b, and Lin28a. Scale bar = 40 µm. n = 3.

The purified cells were then reprogrammed to induced pluripotent stem cells (iPSCs) by adding doxycycline (DOX) to the culture. IPSC colonies appeared approximately 1 week after DOX addition. To establish DOX-independent iPSC clones, we gradually removed DOX from culture and selected for clones that continued to form iPSC colonies. Since the TTF do not express SLC34a1 to drive the expression of Cre recombinase, we introduced exogenous Cre recombinase to excise the loxP-flanked neo cassette to permit the expression of the reprogramming factors. DOX was added to TTF-Cre to initiate reprogramming and was gradually removed to establish DOX-independent TTF iPSC. PTC and TTF iPSCs displayed the typical compact, dome-shaped embryonic stem cell (ESC) colony morphology and they expressed pluripotency-associated markers, Oct4, *Dnmt3b* and *Lin28a* as demonstrated with immunofluorescence microscopy (IF) and RT-qPCR (Fig. 2). Secondary antibody controls showed that secondary antibodies did not bind non-specifically to PTC iPSC in Supplementary Fig. 3). When injected into immunocompromised NOD/SCID mice, all four PTC iPSC lines and all four TTF iPSC lines gave rise to teratomas containing cells from all three germ layers approximately 1 month after injection (Supplementary Fig. 4).

**Figure 3:**
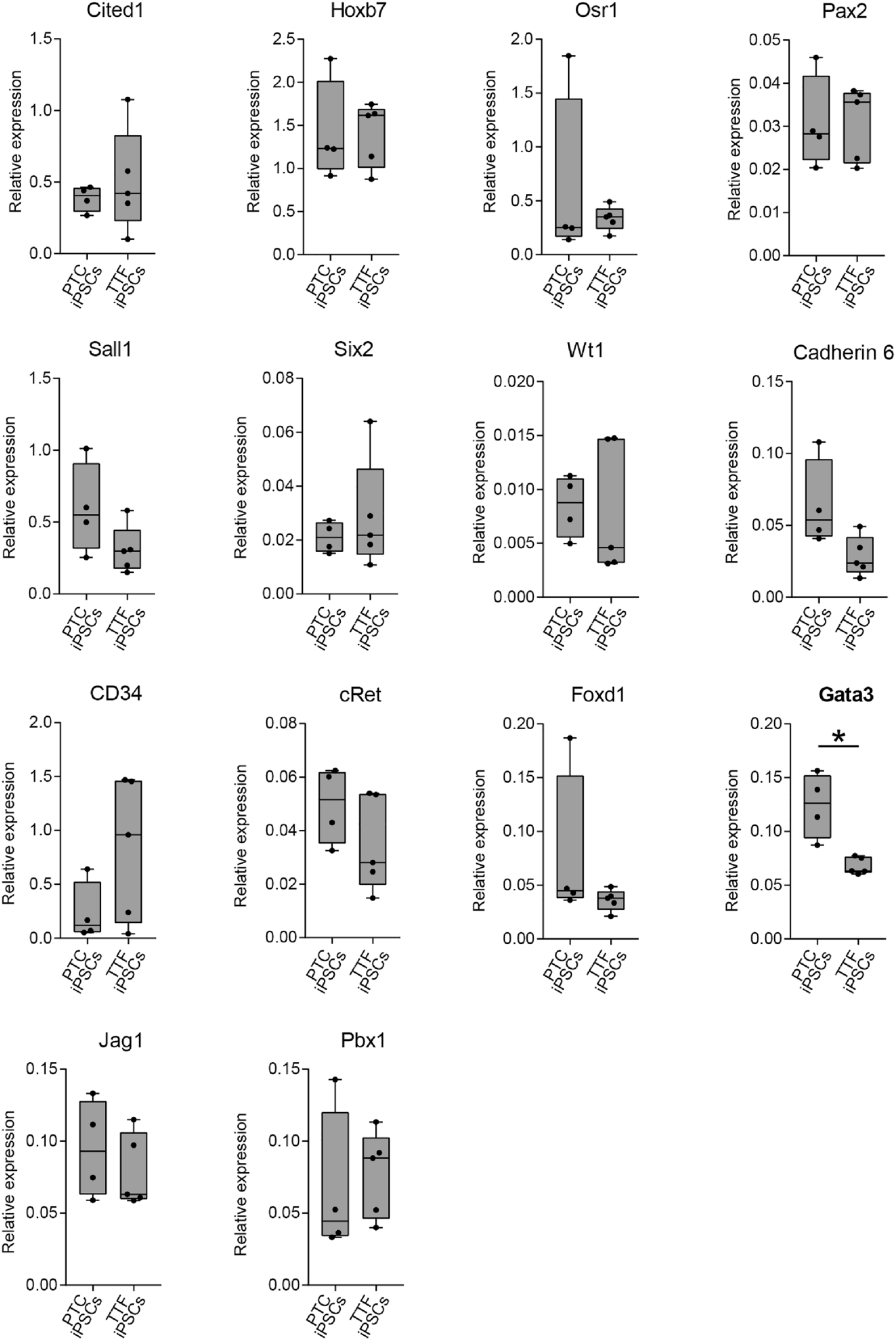
Expression of renal progenitor markers in undifferentiated proximal tubule cell induced pluripotent stem cells and tail tip fibroblast induced pluripotent stem cells. RT-qPCR shows that expression of renal progenitor markers is low in undifferentiated PTC iPSC and TTF iPSC. Y-axis gene expression is relative to E13.5 mouse embryonic kidney which is set at 1.0. Box and whiskers indicate quartiles and maximum/minimum values respectively, with central line showing the median. Asterisk (*) indicates that expression was significantly different (p < 0.05) between PTC iPSCs and TTF iPSCs, as determined by *t* test. n = 4 PTC iPSC and 4 TTF iPSC lines, 3 replicates per line.

To determine if the PTC derived iPSCs had residual expression of renal genes compared to TTF derived iPSCs, we measured expression of several renal progenitor genes by RT-qPCR (Fig. 3).

Expression of the renal genes were low and not significantly different between PTC iPSCs and TTF iPSCs except for *Gata3*, which despite having higher expression levels in PTC iPSCs, showed negligible expression overall. Therefore, IF and qPCR results demonstrate that PTC iPSCs and TTF iPSCs are transcriptionally similar, and this would be expected if reprogramming was equivalent in all cells, no matter the cell type of origin, with each able to reach ground state.

### The epigenetic signature of early passage proximal tubule cell induced pluripotent stem cells and tail tip fibroblast iPSCs

**Figure 4:**
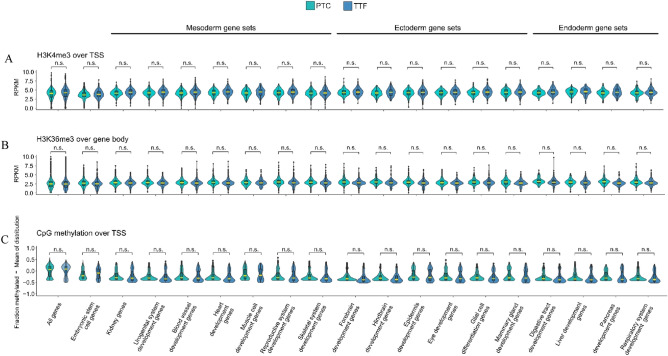
H3K4me3, H3K36me3 landscape of kidney developmental genes and embryonic stem cell (ESC)-associated genes in early passage PTC iPSC and TTF iPSC. Distributions of (**A**) average normalized counts of ChIP-seq reads (RPKM) or (**B**) methylation events normalized to distribution average for PT or TT, for gene sets associated with the indicated system or organ development (refer to Supplementary Table 310.6084/m9.figshare.20368902). Median of each distribution is marked in yellow. Embryonic germ layer of origin is indicated. ANOVA followed by Tukey’s honest significant difference test was used for comparisons. *n.s*. not significant (refer to Supplementary Table 4 for details 10.6084/m9.figshare.20368902).

Although there were no differences observed between PTC iPSCs and TTF iPSCs in terms of renal markers and pluripotency markers, at both the RNA and protein levels epigenetic memory could still be present and have an impact on the differentiation preference of the iPSC lines. To check if PTC and TTF iPSCs show epigenetic differences, we performed ChIP-seq for H3K4me3 (activating), H3K36me3 (elongation) and long-read DNA methylation sequencing on our iPSCs. For DNA methylation, we focused on the transcriptional start site (TSS). Examination of the global histone and DNA methylation patterns showed no significant differences for histone methylation or DNA methylation between TTF-iPSCs and PTC-iPSCs for all genes of the mouse genome (Fig. 4A–C—1st violin plot pair). The analysis was grouped by gene sets for mesoderm, endoderm and endoderm tissues with each germ layer group containing multiple tissue specific gene sets. A specific examination of genes implicated in kidney development and maintenance (Fig. 4A–C—3rd violin plot pair), demonstrated no statistical differences between the PTC and TTF iPSC for histone or DNA methylation of kidney genes. In total we compared 18 Gene Ontology groups including the urogenital system, but no differences were observed in any of the GO groups. Indeed, the global pattern of histone methylation and DNA methylation for multiple mesoderm, endoderm and ectoderm Gene Ontology groups all produced a null result indicating that low passage PTC-iPSCs and the TTF-iPSCs are epigenetically equivalent (Fig. 4A–C).

**Figure 5:**
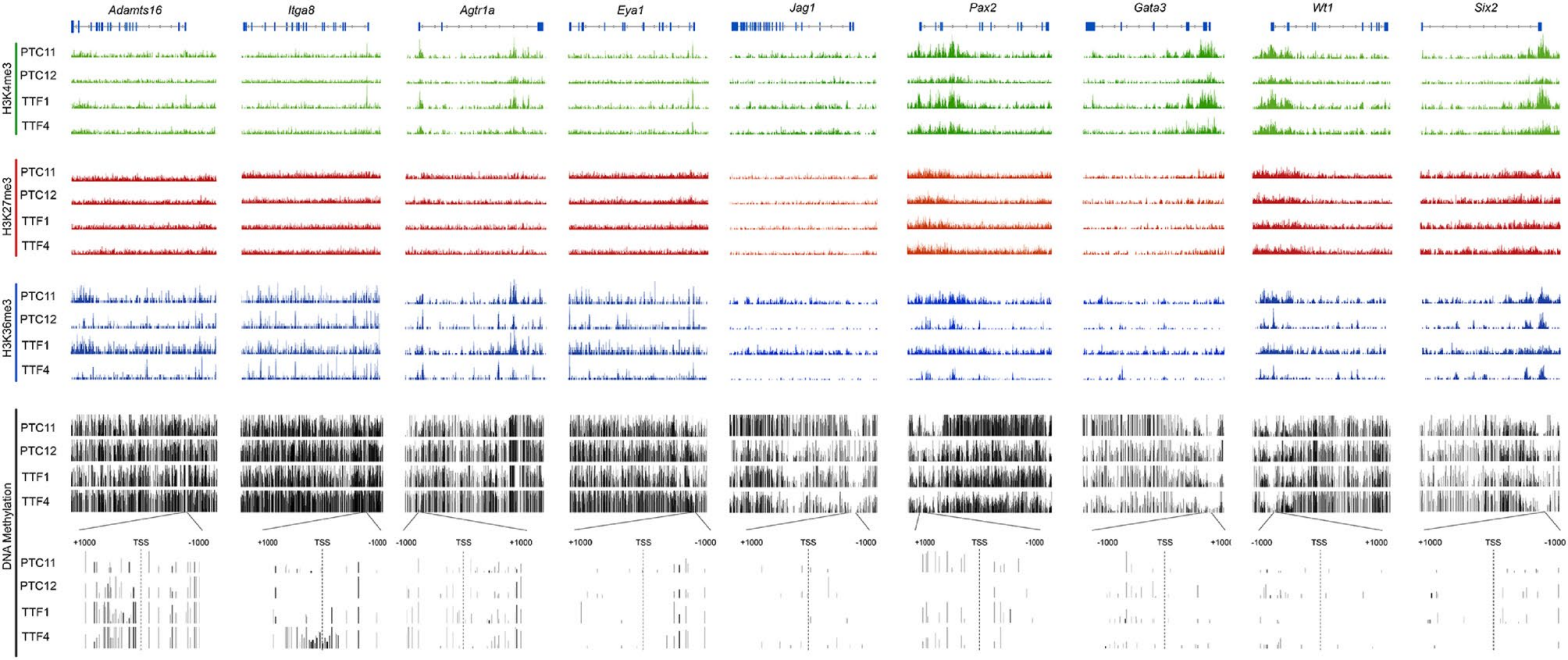
Histone and DNA methylation patterns for renal specific genes verifies no cell of origin bias. Histograms of ChIP-seq read coverage for H3K4me3 (activating), H3K27me3 (repressing) and H3K36me3 (elongation) histone marks, or DNA methylation (as fraction methylated per CpG) over the genomic locus or TSS for renal specific genes Adamts16, Itga8, Agtr1a, Eya1, Jag1, Pax2 Six2, Wt1 and Gata3 show some differences between individual lines but there is no cell of origin pattern observed. For example, PTC11 and TTF1 have similar H3K36me3 content and distribution resulting in more methylation compared to PTC12 and TTF4 which have equal patterns to each other. *TSS* transcription start site.

Nine individual genes implicated in kidney development were chosen for further analysis: (*Adamts16*, *Itga8*, *Agtr1a*, *Eya1*, *Jag1*, *Pax2, Six2, Wt1* and *Gata3* (Fig. 5). Comparison of ChIP-seq for H3K4me3 (activating), H3K27me3 (repressing), H3K36me3 (elongation) and DNA methylation confirmed that there were no differences observed for histone methylation or DNA methylation between PTC-iPSC and TTF-iPSC lines, although individual iPSC line differences could be seen. For example, H3K36me3 methylation for *Jag1* and *Gata3* were higher in PTC11 and TT1 compared to PTC12 and TT4. The DNA methylation pattern within 1000 base pairs of the TSS did demonstrate quantitative differences between different iPSC lines The iPSC line TTF4 has more DNA methylation at the *Itga8* TSS compared to TTF1, PTC11 and PTC12. On the other hand, PTC12 exhibits low levels of DNA methylation around the TSS for Pax2 compared to the other iPSC lines. Generally, we could not detect notable differences in the DNA methylation levels at the TSS between the different iPSC lines for *Adamts16*, *Agtr1a*, *Eya1*, *Jag1*, *Six2, Wt1* and *Gata3*.

### Differentiating proximal tubule cell- and tail tip fibroblast-derived iPSC to renal progenitors

**Figure 6:**
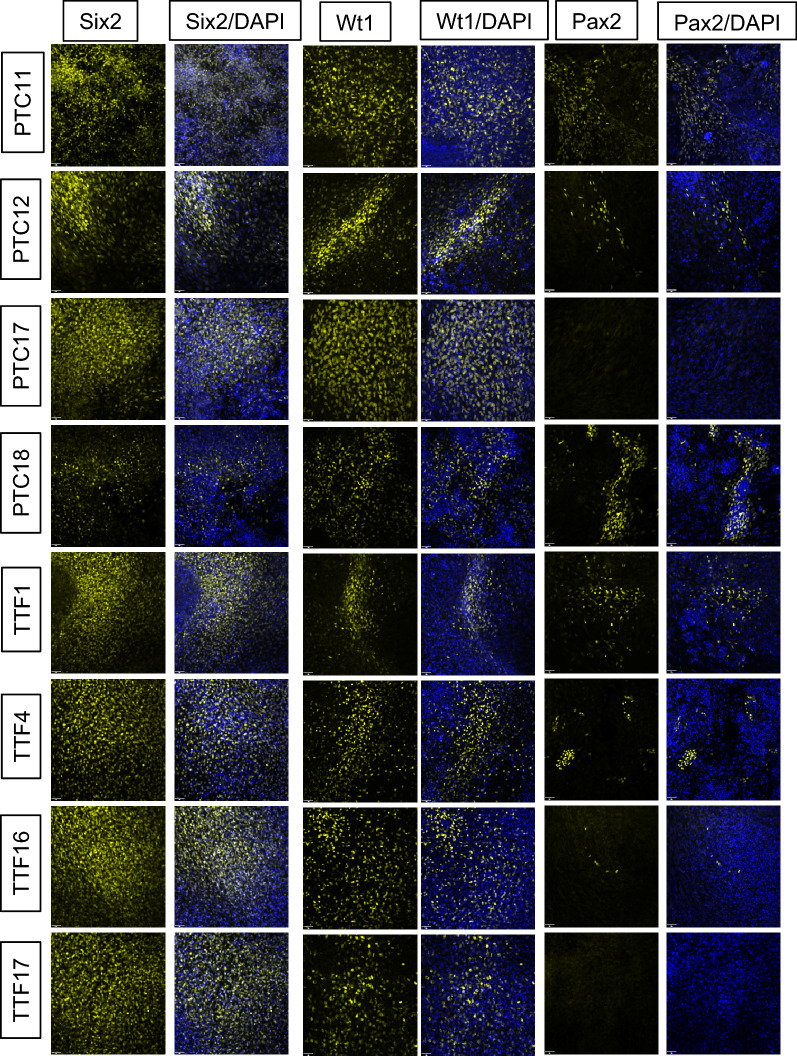
Expression of Six2, Wt1 and Pax2 in proximal tubule cell induced pluripotent stem cells and tail tip fibroblast induced pluripotent stem cells differentiated to renal progenitors. Four PTC iPSC lines and four TTF iPSC lines were differentiated to renal progenitor cells as demonstrated by the presence of the three progenitor cells proteins Six2, Wt1 and Pax2 protein expression. Scale bar = 40 µm.

Analysis of the PTC-iPSC and TTF-iPSC lines indicated similar epigenetic profiles at kidney markers, suggesting that there may be no effect of the cell of origin on epigenetic memory of kidney-derived iPSCs. To test the hypothesis of whether epigenetic memory affects the differentiation capacity of these cells, all eight lines were differentiated to renal cells and analyzed for protein and gene expression. All four PTC iPSC lines and all four TTF iPSC lines were differentiated sequentially to mesoderm (day 2), then to intermediate mesoderm (day 4) and finally to renal progenitor cells (IM/UB) (day 7). We assessed the cells at the final stage (day 7). All eight lines expressed renal progenitor markers Six2 and Wt1 and three PTC iPSC lines and three TTF iPSC lines expressed Pax2 as assessed by immunofluorescence (Fig. 6). Secondary antibody controls can be seen in Supplementary Fig. 5.

**Figure 7:**
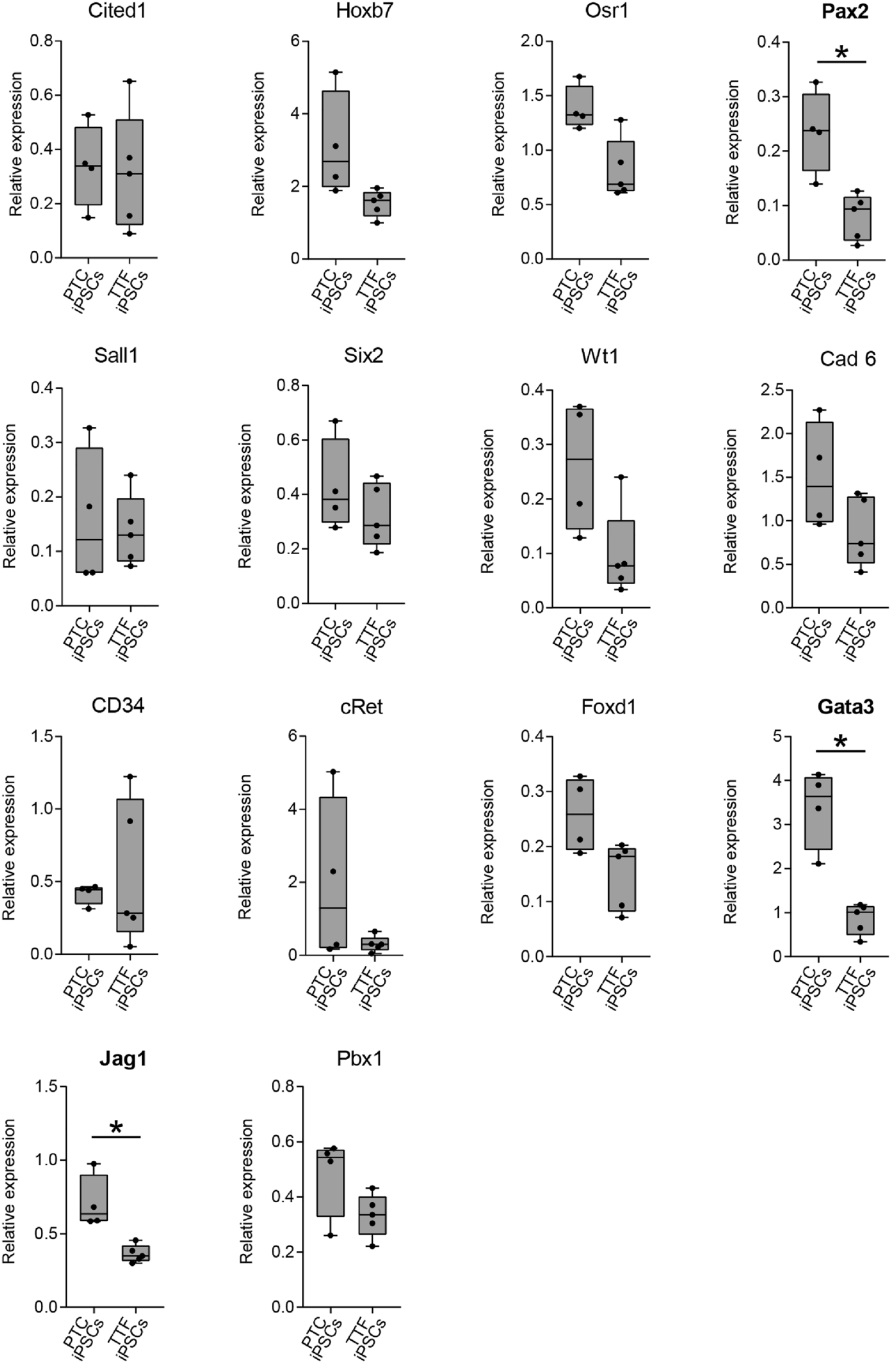
Expression of renal progenitor markers in renal progenitors derived from proximal tubule cell induced pluripotent stem cells (PTC iPSC) compared to renal progenitors derived from tail tip fibroblast induced pluripotent stem cells (TTF iPSC). RT-qPCR shows that Pax2, Gata3 and Jag1 expression are higher in renal progenitors derived from PTC iPSC csmpared to renal progenitors derived from TTF iPSC. Y-axis gene expression is relative to E13.5 mouse embryonic kidney which is set at 1.0. Box and whiskers indicate quartiles and maximum/minimum values respectively, with central line showing the median. Asterisk (*) indicates that expression was significantly different (p < 0.05) from progenitors derived from PTC iPSCs, as determined by *t* test. n = 4 PTC iPSC lines and 4 TTF iPSC lines, 3 replicates per line.

In order to quantitate gene expression and determine if there is a differentiation bias towards renal progenitor cell in the PYTC iPSC we assessed the expression of 14 genes involved in renal development by RT-qPCR (Fig. 7). We investigated the expression levels of metanephric mesenchyme genes: *Osr1, Cited 1, Six2, Sall1, Wt1, Pax2,* UB genes: *Gata3, Jag1, Hoxb7, cRet* and *Cadherin*, endothelial precursor genes: *CD34* and Stromal genes: *Foxd1* and *Pbx1* in the four PTC iPSC lines and the four TTF iPSC lines when differentiated to renal progenitor cells. Three of the fourteen genes assessed, *Gata3*, *Jag1* and *Pax2*, had significantly higher expression levels in the PTC iPSC group. The remainder were not significantly different. Despite having higher expression levels these three genes did not show any epigenetic differences between the PTC iPSCs or TTF iPSCs lines.

## Discussion

The ability of patient derived cells to undergo reprogramming followed by differentiation to a specific cell or tissue type has great potential for treating disease. Being able to produce patient matched stem cells and therefore patient matched therapeutic cells or organs for transplant resolves a major issue in donor organ/tissue procurement and transplant success. The success of producing differentiated cells from pluripotent cells has been achieved by many labs but the efficiency varies widely. The differentiation frequency can be low and requires enrichment by removal of non-differentiated cells through fluorescent activated cell sorting (FACS), columns or selective medium that supports only the target cells. Epigenetic memory offers a way to increase the efficiency of differentiation. We are interested in developing cell based therapies for kidney disease and since renal cells can be isolated from the urine, determining the epigenetic memory status of renal cells has important clinical applications.

There are multiple explanations given for the epigenetic differences observed between different iPSC lines, such as donor genome differences, reprogramming methods, incomplete reprogramming, and cell of origin differences. During embryo development, adequate control of the DNA methylation pattern is critical to ensure normal fetal development^29^. Conversely, removal of the adult methylation pattern during somatic cell reprogramming is also crucial to ensure pluripotency is achieved. With epigenetic data available on hundreds of iPSC and human embryonic stem cells (ESC) lines, a comparison revealed there is a large common gene set observed between iPSC and ESC^30,31^. But when individual iPSC lines were compared to each other they did demonstrate differences. Importantly, studies revealed that even with seemingly fully reprogrammed lines there were ‘cell of origin’ differences observed at the epigenetic level that could influence the differentiation capacity of the iPSC lines. Experimental variables can also account for epigenetic differences between cell lines, so in order to determine if there is epigenetic memory in renal cells while controlling for as many technical variables as possible we developed a quadruple transgenic mouse in order to determine if there is epigenetic memory in renal cells. Understanding the impact that genetic and epigenetic differences could have on reprogramming and differentiation is important for determining the feasibility of using iPSCs for clinical therapies.

Epigenetic variability due to cell of origin is referred to as epigenetic memory and is defined as epigenetic patterns unique to the cell of origin that can bias differentiation of the resultant iPSC towards the cell of origin. The investigation of epigenetic memory by multiple labs and its impact on the differentiation capacity of the iPSC line has produced mixed results depending on the tissue being investigated suggesting that epigenetic memory may not be universal for all cells. Heslop et al.^32^, using human hepatocytes, found there was no epigenetic memory, while Bar-Nur et al.^5^ using human islets showed epigenetic memory in early passage iPSC lines. One group using isogenic fetal tissue to make iPSC lines and then comparing the DNA methylation patterns to the original human fetal tissue, determined that only one of six tissues investigated, brain tissue, demonstrated evidence of epigenetic memory^33^. In another study comparing isogenic embryonic stem cells, iPSCs and somatic cells (fibroblasts, retinal pigment epithelium and neural cells) derived from the pluripotent lines, Shutova et al. could not detect any epigenetic memory as defined by similar methylation patterns of CpGs in the somatic cells and the derived iPSC lines. Similar to our results they observed a small number differentially expressed genes indicative of somatic memory but this did not correlate with CpG methylation sites. The disconnect between gene expression and epigenetic regulation was not explained but they did observe that iPSC lines can carry a somatic cell signature of a previous ‘general’ differentiation state but the somatic signature is not specific to the cell of origin^34^.

Focusing on reprogrammed mouse proximal tubule cells (PTC) and tail tip fibroblast (TTF) from a reprogrammable mouse helped to reduce the influence of individual donor genetic diversity and reprogramming methodologies on iPSC differentiation. The reprogrammable mouse/secondary reprogramming system is a system in which the mice have copies of drug-inducible reprogramming factors inserted into the genome. Hence, all cells isolated from the reprogrammable mouse have the same copies of reprogramming genes inserted into the same location in the genome. This is important when evaluating differences between iPSC lines as transgene dose and genome location could affect the type of iPSCs generated and their differentiation propensity^35^.

The quadruple transgenic mouse model also allowed us to fluorescently mark mature PTC. It was important for us to fluorescently label PTC because primary PTC grown on conventional 2D cultures have a tendency to de-differentiate and lose renal marker expression, making them difficult to identify in culture^36^. Proximal tubule cells isolated from the SLC34a1-GCE; tdTomato; rtTAROSA (neo-in); OKMS-250 mice have three fluorescent reporters: green fluorescence from the Cre recombinase, tdTomato, and mCherry from the OKMS transgene. As described in the methods, we sorted for PTC using FACS. The FACS purified PTC were then cultured in DOX to initiate reprogramming to iPSC. TTF cells were transfected with exogenous Cre recombinase because they do not express SLC34a1-GCE. We used a non-integrating episomal Cre recombinase, which ensured that genes outside of the loxP-flanked regions were not altered during Cre-mediated recombination. We chose TTF as our control cells because they are easy to isolate and are well studied as fibroblasts are commonly used in primary and secondary reprogramming studies^1,37–39^.

In our study we did not observe higher renal gene expression in the PTC iPSCs compared to the TTF iPSCs. The gene expression patterns of the TTF iPSCs and the PTC iPSCs were similar indicating that there was no difference between the iPSC lines. Since epigenetic memory can be observed in DNA methylation patterns and histone modification patterns that have some similarity to the cell of origin we investigated the global DNA methylation and histone modifications, including repression, activating and poised markers. Our data revealed no differences due to cell of origin. Close examination of histone modifications of a sub group of renal genes gave the same result.

Interestingly, upon differentiation of the TTF iPSC lines and PTC iPSC lines to renal progenitor cells we did observe that the kidney progenitors derived from PTC iPSCs expressed higher levels of kidney progenitor genes *Pax2, Gata3* and *Jag1* compared to the TTF-derived iPSCs. Since there were no epigenetic differences between the PTC iPSCs and TTF iPSCs the data indicates that higher renal gene expression in PTC-iPSCs derived renal progenitor cells is not due to epigenetic memory and other mechanisms may be in play.

Preferential differentiation of iPSCs based on cell of origin studies have revealed that the basis for the differentiation bias can be due to different mechanisms. We saw no epigenetic differences in the undifferentiated iPSC lines but did observe unequal gene expression for three genes during differentiation. Polo et al.^8^ showed that there were residual cell-specific gene expression of mature genes in the undifferentiated iPSCs they were investigating (muscle versus granulocytes) and found that when they interrogated gene activating histone acetylation marks at the promoter regions for granulocyte genes, *Lysozyme and Gr-1* and muscle genes, *Cxcr4, Itgb1*, they observed differences that could account for genes expression differences in the iPSCs indicating a cell of origin memory. Furthermore, they found that the largest differences observed were for histone modifications and not DNA methylation and the differences were due to cell of origin and not donor differences. In contrast, in our study we observed differences in DNA methylation around the promoter sites between individual iPSC lines regardless of cell of origin.

Our data, within the context of other studies, indicates that epigenetic memory is not universal and maybe cell specific. Secondary reprogrammable systems used in this study and Polo et al., along with the ability to confirm the cell being reprogrammed through genetic reporters, helps to remove technical variables that could result in observable differentiation and gene expression bias.

In this study we controlled for many confounding factors and demonstrated that the DNA and histone methylation patterns are not statistically different for inter and intra line comparisons. Despite observing that three renal specific genes were upregulated in the renal progenitors differentiated from the PTC lines compared to the TTF lines, our data concludes that epigenetic memory is not the source of the possible bias in differentiation.

## Supplementary Information

Supplementary Figures.

Supplementary Table 1.

Supplementary Table 2.

Supplementary Table 3.

Supplementary Table 4.

## Data Availability

Long-read DNA sequencing and ChIP-seq datasets generated in this study Bam and BIGWIG files are available on the GEO public database under accession number GSE195511 and can be accessed at the following link: https://www.ncbi.nlm.nih.gov/geo/query/acc.cgi?acc=GSE195511. To access it, the webpage requires the following secure token: cdunaokebfsbtct. Supplementary Tables 1–4 are available in the found in the Figshare data base: 10.6084/m9.figshare.20368902.
