# Supplementary material for: Determining epigenetic memory in kidney proximal tubule cell derived induced pluripotent stem cells using a quadruple transgenic reprogrammable mouse
Source: Sci Rep. 2022 Nov 25;12:20340. doi: 10.1038/s41598-022-24581-z (PMC9700797; doi:10.1038/s41598-022-24581-z)
Supplement: Supplementary file 1 — Supplementary Figures. [file 41598_2022_24581_MOESM1_ESM.pdf]

Supplementary Figure 1

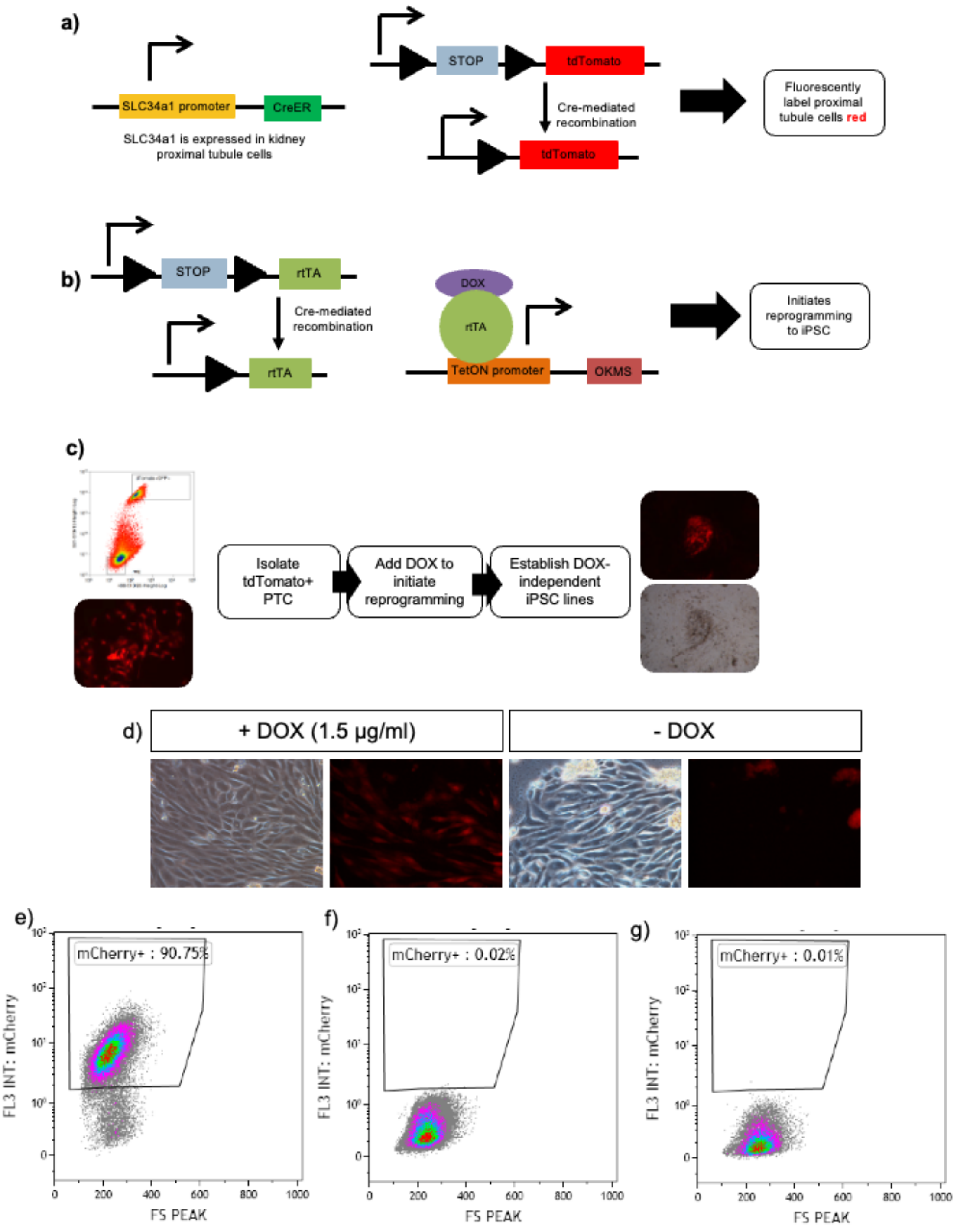

Supplementary Figure 1: Reprogramming proximal tubule cells from SLC34a1-GCE; tdTomato; rtTAROSA (neo-in); OKMS-250 to induced pluripotent stem cells (iPSC). a) The expression of Cre recombinase is under the control of the SLC34a1 promoter, which is active in mature proximal tubule cells. The Cre recombinase-mediated excision of the loxP-flanked STOP sequence allows the expression of mCherry. **b)** Cre recombinase-mediated excision also allows for the expression of rtTA. In the presence of rtTA and DOX, the TetON promoter is activated, driving the expression of the reprogramming factors (Oct4, Klf4, Myc, and Sox2). **c)** tdTomato+ proximal tubule cells were sorted using fluorescence activated cell sorting (FACS). Once isolated they were reprogrammed to induced pluripotent stem cells (iPSC) by the addition of doxycycline (DOX). **d)** Cells from the rtTAROSA (neo-in); OKMS-250 mouse cells prior to breeding with the SLC34a1-GCE; tdTomato mice were tested to determine if mCherry expression only occurred upon DOX induction. Fluorescent images demonstrate mCherry appears only in the DOX treated cells. Flow cytometry further confirms that mCherry is present in **(e)** cells treated with DOX and absent in **f)** untreated cells and **g)** wildtype cells.

Supplementary Figure 2

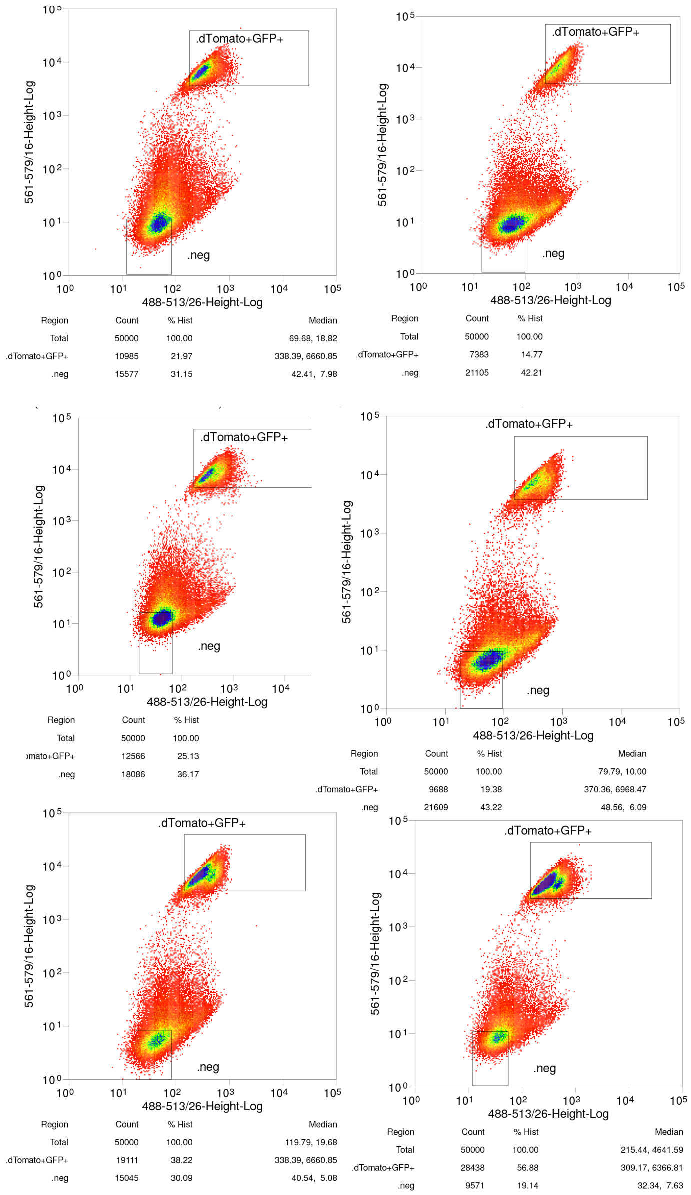

Supplementary Figure 2: Isolation of GFP+/tdTomato+ cells

GFP+/tdTomato+ proximal tubule cells isolated from the quadruple transgenic mouse: 29% range 15-56% n=6

Supplementary Figure 3

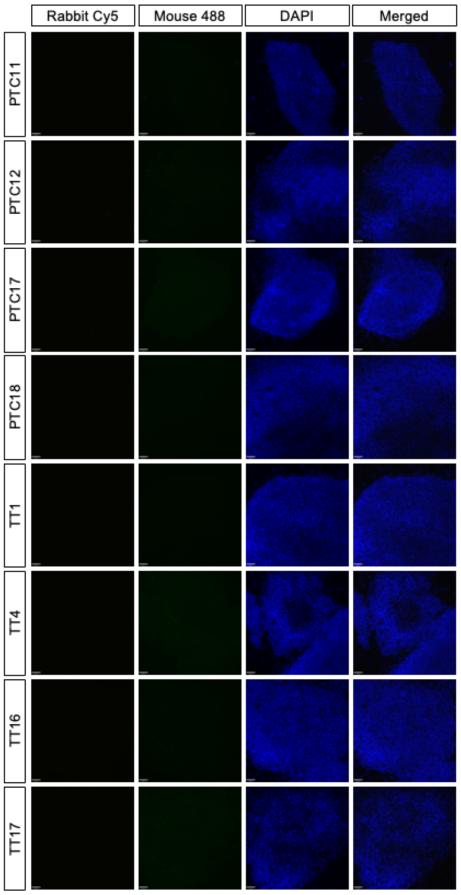

Supplementary Figure 3: Secondary antibody controls for undifferentiated proximal tubule cell induced pluripotent stem cells (PTC iPSC) and tail tip fibroblast (TTF) iPSC. Immunocytochemistry shows that secondary antibodies do not bind non-specifically to undifferentiated PTC iPSC. Scale bar = 40 μm.

Supplementary Figure 4

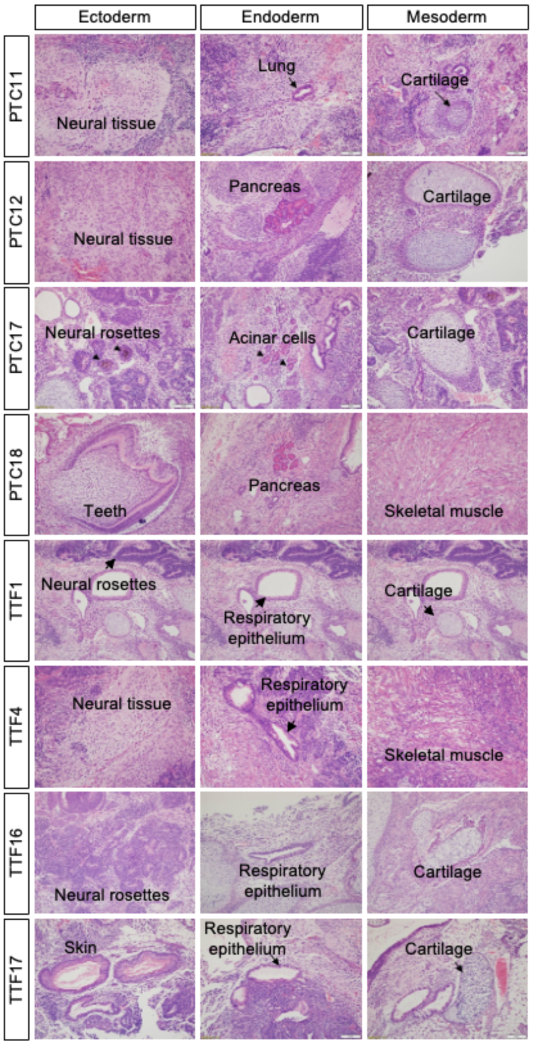

Supplementary Figure 4: Proximal tubule cell induced pluripotent stem cell (PTC iPSC) and tail tip fibroblast (TTF) iPSC form teratomas in immunocompromised NOD/SCID mice. Hematoxylin and eosin (H&E) staining reveals that PTC iPSC and TTF iPSC injected into NOD/SCID mice give rise to teratomas containing tissues from all three germ layers. Images were taken with a 20x objective.

Supplementary Figure 5

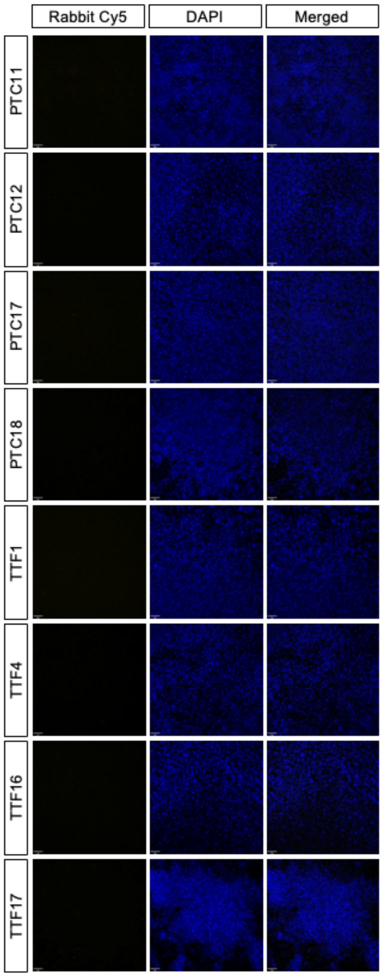

Supplementary Figure 5: Secondary antibody controls for proximal tubule cell induced pluripotent stem cells (PTC iPSC) and tail tip fibroblast (TTF) iPSC differentiated to kidney progenitor cells. Immunocytochemistry shows that secondary antibodies do not bind non-specifically to undifferentiated PTC iPSC. Scale bar = 40 μm.
